# A case of severe Aicardi–Goutières syndrome with a homozygous *RNASEH2B* intronic variant

**DOI:** 10.1038/s41439-024-00291-y

**Published:** 2024-08-26

**Authors:** Yuri Shibata, Akimichi Shibata, Takeshi Mizuguchi, Naomichi Matsumoto, Hitoshi Osaka

**Affiliations:** 1 Department of Pediatrics, Sano Kosei General Hospital, Tochigi, Japan; 2 Department of Pediatrics, Jichi Medical University, Tochigi, Japan; 3 Department of Pediatrics, Japanese Red Cross Ashikaga Hospital, Tochigi, Japan; 4 Department of Human Genetics, Yokohama City University Graduate School of Medicine, Kanagawa, Japan

**Keywords:** Medical genetics, Immunological disorders

## Abstract

We report a case of severe Aicardi–Goutières syndrome caused by a novel homozygous *RNASEH2B* intronic variant, NC_000013.10(NM_024570.4):c.65-13G > A p.Glu22Valfs*5. The patient was born with pseudo-TORCH symptoms, including intracranial calcification, cataracts, and hepatosplenomegaly. Furthermore, the patient exhibited profound intellectual impairment and died at 14 months due to aspiration pneumonia accompanied by interstitial lung abnormalities. The severity of the patient’s symptoms underscores the critical role of the C-terminal region of RNase H2B.

Aicardi–Goutières syndrome (AGS), a rare progressive encephalopathy caused by excessive upregulation of interferon (IFN) α activity, is the first identified type I interferonopathy; type I interferonopathies are a group of Mendelian autoimmune and autoinflammation disorders characterized by pathogenic polymorphisms that upregulate type I IFN signaling^1^. *RNASEH2B* encodes a subunit of the RNASEH2 complex, a conserved DNA repair enzyme that excises incorrectly inserted ribonucleotide monophosphates^2^. Pathogenic *RNASEH2* variants lead to the accumulation of DNA repair metabolites that stimulate the cyclic GMP–AMP synthase (cGAS)–stimulator of interferon genes (STING) pathway, resulting in the overproduction of type I IFNs^2^. The excessive autoimmune response causes inflammation without infection, presenting as symptoms of congenital infections caused by *Toxoplasma gondii*, rubella virus, cytomegalovirus, and herpes simplex virus types 1 and 2 (TORCH syndrome) in prenatal-onset AGS^1^. All reported AGS patients are heterozygous for the pathogenic variant c.65-13G > A in *RNASEH2B*, the gene most commonly mutated in patients with AGS. However, the effect of the c.65-13G > A variation is not known, and the clinical course was detailed in only one patient.

The patient was the third child born to nonconsanguineous Sri Lankan parents (Fig. 1a). Both parents and two siblings were asymptomatic. Severe fetal growth restriction and microcephaly were notable beginning in the second trimester of pregnancy. The infant was born via emergency cesarean section due to fetal bradycardia at 38 weeks and 4 days of gestation. The birth weight was 1706 g (−3.89 SD), the head circumference was 27.0 cm (−4.37 SD), and the Apgar scores were 7 and 8 points at 1 and 5 min, respectively. Notably, respiratory impairment was apparent from birth, and tracheal intubation was performed. Ventilator management was continued for 5 days. The high-flow nasal cannula was able to be removed at 40 days, and oxygen treatment was stopped at 98 days of age. A chest computed tomography scan revealed bilateral air-space diffuse opacities. Elevated levels of Krebs von den Lungen-6 (KL-6) (496 U/ml; normal range <250 U/ml) and surfactant protein-D (SP-D) (149 ng/ml; normal range <110 ng/ml) were detected, which indicated interstitial lung disease. The infant also had feeding difficulties and diarrhea, accompanied by electrolyte imbalance and metabolic acidosis. She was able to gain weight through tubal feeding of hydrolyzed formula. During the first few months, hyperinsulinemic hypoglycemia (insulin levels of 4.93–15.50 μU/ml with low blood glucose concentrations of 33–47 mg/dl) was controlled with continuous intravenous administration of glucose and tubal feeding of cornstarch, and iron deficiency anemia was treated with red blood cell transfusion and iron medication. As antimicrobial prophylaxis, cefaclor (10 mg/kg/day) was started for bilateral hydronephrosis and continuous afebrile pyuria. Cataracts, deafness, and hepatosplenomegaly were also observed, with elevated levels of transaminases (aspartate aminotransferase, 47–212 U/l; alanine aminotransferase, 76–287 U/l). Chilblain-like lesions appeared on the toes at approximately 2 months of age (Fig. 1b). Neuroimaging of the brain revealed intracranial calcification and polymicrogyria (Fig. 1c, d). Generalized clonic seizures were observed mainly in the first several weeks and improved with phenobarbital administration. Interventricular septal hypertrophy (interventricular septum at end diastole, 8.95 mm; z = 3.96) was observed from 6 months of age, although cardiac function remained normal without treatment. The patient developed recurrent aspiration pneumonia and died at 14 months of age from markedly exacerbated respiratory failure and an increased inflammatory response. Dystonia and spasticity were severe, and the AGS score, a composite score for neurological function calculated based on 11 key symptoms of AGS, was 0 throughout the patient’s life, with no head control, social smile, or vocalizations^3^.

**Fig. 1:**
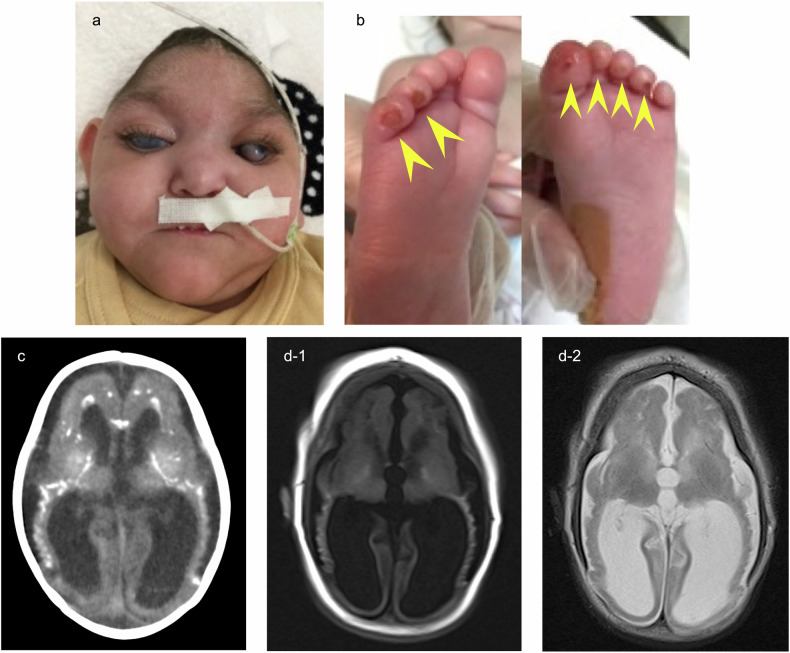
Patient details. **a** Patient at 12 months of age with microcephaly and bilateral cataracts. **b** Chilblain-like lesions (yellow arrowheads) at 2 months of age. **c** Brain CT at 1 month of age showing calcification of the basal ganglia and white matter. **d** Brain MRI (**d-1** T1-weighted, **d-2** T2-weighted) at 4 months of age revealed cerebral atrophy and polymicrogyria.

Although TORCH syndrome was suspected, serologies for toxoplasmosis, rubella, cytomegalovirus, and herpes infections were negative. The cerebrospinal fluid was not examined; however, the patient met the five main diagnostic criteria necessary to establish a clinical diagnosis of AGS: (i) early onset encephalopathy with psychomotor delay, spasticity, extrapyramidal signs, and microcephaly; (ii) calcifications visible particularly at the basal ganglia level but also extending to the periventricular white matter; (iii) cerebral white matter abnormalities; (iv) cerebral atrophy; and (v) exclusion of pre/perinatal infections, particularly the TORCH complex^4^. At 3 months of age, panel sequencing for hereditary autoimmune diseases was performed at the Kazusa DNA Research Institute, and seven of the nine genes—*RNASEH2A, SAMHD1, RNASEH2B, RNASEH2C, TREX1, IFIH1*, and *ADAR—*known to harbor mutations associated with AGS were analyzed. Although a homozygous missense variant was detected in *RNASEH2B* (NC_000013.11(NM_024570.4):c.895A > G), the variant was classified as likely benign according to the American College of Medical Genetics and Genomics (ACMG)/Association of Molecular Pathology (AMP) classification^5^. Research-based whole-exome sequencing revealed another homozygous intronic variant in *RNASEH2B*, NC_000013.10(NM_024570.4):c.65-13G > A, p.Glu22Valfs*5^6^, which was classified as pathogenic (PVS1 + PS3 + PM2) according to the ACMG/AMP guidelines. Family trio-based whole-exome sequencing confirmed that this variant was inherited from both the heterozygous father and the mother (Fig. 2). No other pathogenic/likely pathogenic variants were detected. Therefore, the patient was diagnosed with AGS due to the presence of a pathogenic variant of *RNASEH2B*. This study was approved by the Ethics Committee of Jichi Medical University (approval number, A22-022), and written informed consent was obtained from the parents.

**Fig. 2:**
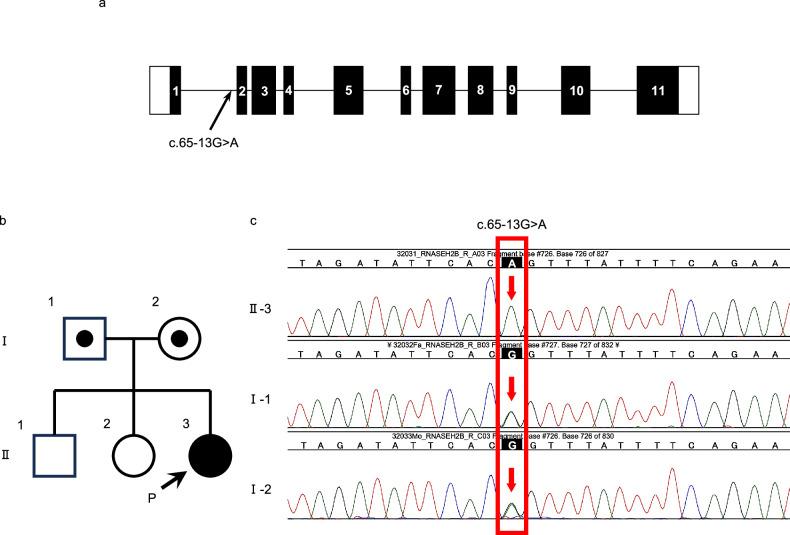
Genetic analysis of the family. **a** Schematic representation of the *RNASEH2B* gene, with the position of the identified mutation. The colored boxes with numbers represent exons, and the horizontal lines represent introns. **b** Family pedigree. **c** Trio-based clinical genome sequencing showing the c.65-13G > A variant in *RNASEH2B*. DNA sequence electrophoretograms of the homozygous proband and heterozygous parents.

In genotype–phenotype correlations for AGS, prenatal onset is mostly associated with *TREX1* variants, whereas *RNASEH2B* variants are associated with infantile or later-onset patients^7^. *RNASEH2B* is usually the most commonly mutated gene in patients with AGS (36–59%) and is associated with lower morbidity and mortality rates than other genotypes^8,9^. The pathogenic intronic variant c.65-13G > A has been described only as a heterozygous variant in a few studies^6,8,10^. The prevalence of this variant is 0.000003984 (1/251008) in the Genome Aggregation Database^11^, with a predicted protein change of p.Glu22Valfs*5. The c.65-13G > A variant has been shown to cause aberrant splicing, resulting in the retention of 11 nucleotides, which subsequently introduces a frameshift^10^. Additionally, the transcript may undergo nonsense-mediated mRNA decay due to the introduction of a stop codon. Rice et al.^6^ reported three patients, Crow et al.^8^ reported three families, and Garau et al.^10^ reported one patient who presented with multiple neurological symptoms at 12 months of age (Table 1). All these reported cases harbored compound heterozygous mutations, and the effect of c.65-13G > A is not known. The clinical course was detailed in only one case that progressed gradually compared with our case with homozygous variation^10,12^. The splice variant in intron 1, c.65-13G > A, is presumed to result in the loss of an amino acid region at the interface between RNase H2B and RNase H2C that strongly destabilizes the entire heterotrimeric complex^10^. Western blotting analysis revealed a reduction in the levels of all RNase H2 subunits and a decrease in RNase H2B expression^10,13^. Although the RNase H2A subunit harbors the catalytic core of RNase H2, all three subunits are required for its activity. Whereas the precise roles of the RNase H2B and RNase H2C subunits are not well understood, a functional proliferating cell nuclear antigen-interacting protein motif in RNase H2B directs RNase H2 activity in replication and repair^14^.

**Table 1:** Details of reported cases with c.65-13G > A variant.

	Age at report (months)	Sex	Ethnicity	Mutations	Clinical manifestations
Patient 1^6^	66	F	White French	c.65-13G > A p.Glu22Valfs*5/c.529G > A p.Ala177Thr	NA
Patient 2^6^	9	M	White French	c.65-13G > A p.Glu22Valfs*5/c.529G > A p.Ala177Thr	NA
Patient 3^6^	3	F	French European	c.65-13G > A p.Glu22Valfs*5/c.529G > A p.Ala177Thr	NA
3 families^8^	NA	NA	North African, Northern European	c.65-13G > A p.Glu22Valfs*5/NA	NA
Patient 4^10,12^	12, 43	M	Indian mother Italian father	c.65-13G > A p.Glu22Valfs*5/c.253C > G p.L85V	Late onset and slowly progressive: presented with irritability, sleep disturbances, and neuromotor regression.
Present case	Deceased at 14	F	Sri Lankan	c.65-13G > A p.Glu22Valfs*5/c.65-13G > A p.Glu22Valfs*5	Prenatal onset with multiple pseudo-TORCH symptoms.

*NA* not applicable.

The pathophysiology of RNase H2 mutations leading to a disease phenotype is not completely known. RNase H2-deficient mice exhibit significant DNA damage, which is usually lethal in the embryonic stage^2,13^. Congenital TORCH-like symptoms were reported in only three patients with the *RNASEH2B* genotype^9,15^: p.A177T/p.Ex9_Ex11del, p.V185G/p.V185G, and c.322-3C > G/c.322-3C > G. The patient with a homozygous intronic mutation of c.322-3C > G exhibited pseudo-TORCH symptoms similar to those observed in our case^15^. The younger sibling of this patient also presented profound microcephaly and fetal hydrops, and the pregnancy ended with intrauterine fetal death at gestational week 35. Reverse transcription‒polymerase chain reaction revealed skipping of exon 5 and generation of an out-of-frame transcript. Thus, the homozygous intronic mutations c.322-3C > G and c.65-13G > A (identified in this study) are associated with the most severe AGS phenotype, indicating the essential function of the C-terminal amino acid region of RNase H2B. The asymptomatic parents of the siblings with the homozygous intronic mutation c.322-3C > G were related five generations ago^15^. Considering the rarity of the c.65-13G > A variant, there may be a possibility that the parents in our case were also distantly related to each other in Sri Lanka.

In summary, a homozygous pathogenic intronic mutation, c.65-13G > A, in *RNASEH2B* was reported for the first time in a patient with prenatal-onset severe AGS. This case illustrates the crucial role of the C-terminal amino acid region of RNase H2B in AGS pathogenesis.

## HGV Database

The relevant data from this Data Report are hosted at the Human Genome Variation Database at 10.6084/m9.figshare.hgv.3424.
